# Real-World Lessons with Fremanezumab as the Third Available CGRP Monoclonal Antibody in a Third-Level Hospital: Focus on the Factors Predicting Response

**DOI:** 10.3390/jcm14041054

**Published:** 2025-02-07

**Authors:** Marcos Polanco, Gabriel Gárate, Julia Sánchez-Gudín, Jorge Madera, Julio Pascual, Vicente González-Quintanilla

**Affiliations:** 1 Service of Neurology, University Hospital Marqués de Valdecilla, Universidad de Cantabria and IDIVAL, Av. Valdecilla s/n, 39008 Santander, Spain; marcosarsenio.polanco@scsalud.es (M.P.); gabriel.garate@gmail.com (G.G.); jorge.madera@scasalud.es (J.M.); julio.pascual@scsalud.es (J.P.); 2 Service of Pharmacy, University Hospital Marqués de Valdecilla, Av. Valdecilla s/n, 39008 Santander, Spain; julia.sanchez@scsalud.es

**Keywords:** CGRP antibodies, chronic migraine, fremanezumab, high-frequency episodic migraine

## Abstract

**Background:** Fremanezumab was the third CGRP antibody available in our hospital. This examination of our experience with fremanezumab is focused on identifying the predictors of response. **Methods:** This was a prospective observational study in which we included high-frequency episodic/chronic migraine (HF/CM) patients who were prescribed fremanezumab during the year 2023. Our research involved collecting data on their demographic details, diagnoses made, treatments received, prophylactic measures taken in the past, and any comorbid conditions present. The number of headaches was documented for one quarter prior to and after the initiation of fremanezumab. **Results:** Eighty-nine patients received fremanezumab (86.5% female, 45.8 ± 12.5 years old, 70.1% naive). The headache days decreased from 21.1 ± 7.6 to 12.4 ± 11.2 days during the initial three months of the treatment, and a total of 55 patients (61.8%) exhibited a response rate of ≥50%. Six out of ten patients refractory to erenumab for at least 6 months responded to fremanezumab. Totals of 17 and 26 patients had been treated at least with galcanezumab or erenumab. The elements influencing non-response were as follows: prior failure to respond to both erenumab and galcanezumab (*p* < 0.0001), HF/CM length (11.9 ± 7.1 years in non-responders vs. 5.8 ± 4.8 in responders; *p* < 0.001), the presence of fibromyalgia (*p* < 0.001), anxiety–depression (*p* < 0.001), an almost daily headache baseline (>28 days/month) (*p* < 0.0001), and analgesic overuse (*p* < 0.0001). The response rate was unaffected by age and experience. After a multivariate logistic analysis, almost daily headaches (*p* < 0.001), a length of HF/CM > 6 years (*p* = 0.015), and anxiety–depression (*p* = 0.017) remained significant. Fremanezumab showed excellent tolerance. **Conclusions:** These real-life results confirm the efficacy of fremanezumab. The main factors associated with a lack of response were almost daily/daily headaches and a disease duration > 6 years. Half of the patients who failed to respond to erenumab improved on fremanezumab, making it sensible to switch to a treatment with a different mechanism of action, but trying a third anti-CGRP treatment in patients with no response to both a receptor-targeted and a ligand-targeted CGRP antibody hardly seems justifiable from our experience.

## 1. Introduction

Fremanezumab, a monoclonal antibody against the calcitonin-gene-related peptide (CGRP) ligand, was the third CGRP antibody approved for migraine prevention following the launch of erenumab (against the CGRP receptor) and galcanezumab, with which it shares a mechanism of action. Extensive investigations in phase 2–3, randomized, placebo-controlled clinical trials have shown its clear efficacy, with excellent tolerability and no new safety concerns in patients with episodic migraines [1], high-frequency episodic migraine (HF) [2], and chronic migraine (CM) [3,4,5] and in difficult-to-treat migraine patients unresponsive to up to four classes of preventive medications [6]. Clinical trials, however, test drugs under the ideal conditions, and real-life studies are necessary to further explore their effectiveness, safety, and tolerability and to test new hypotheses in unselected patients in routine circumstances. Several real-world reports have confirmed the clinical effectiveness of and adherence to fremanezumab in patients with migraine in different populations, including those with refractory migraine [7,8,9,10,11,12]. Nevertheless, around 40% of migraine patients remained unresponsive to CGRP antibodies, including fremanezumab, in these studies. Considering the current absence of a reliable disease biomarker [13,14], it would be important to identify clinical predictors of a response to optimize the clinical outcomes and resource allocation. A few real-world studies have suggested that certain baseline demographics and phenotypic characteristics could help with predicting responsiveness to CGRP antibodies. Even though this issue still remains open, as the results from the available studies are heterogeneous, there is some consensus that adhering to typical migraine characteristics (e.g., unilateral, periocular, episodic pain) is associated with a better response to CGRP antibodies [15,16,17,18,19,20].

Fremanezumab was made commercially available in Spain at the end of 2020, while reimbursement in our health region arrived in 2022 for patients with HF or CM who had previously failed to respond to at least three preventatives, including onabotulinumtoxin type A in the case of CM patients. Our objective in this prospective study was to further analyze, in a real-world population of HF/CM patients, the predictive role of demographics, certain clinical/phenotypic factors, and the main comorbidities in the response to fremanezumab.

## 2. Materials and Methods

This was a prospective observational study at our Headache Clinic. Eligible participants were those who had been prescribed fremanezumab due to HF/CM during the year 2023. Inclusion in the analysis required at least 1 injection of fremanezumab. Patients were asked to complete a standard headache diary as a routine part of their migraine management. According to the regulations in our country, to be treated with a CGRP antibody, all migraine patients had at least 8 headache days per month and failed to respond to at least three classical oral preventatives, plus onabotulinumtoxin type A (two treatments; at least one with 200 U) in cases of CM.

Patients were seen in the clinic one month after the first injection of fremanezumab and again after the third month of treatment. Prior to the administration of fremanezumab, we collected data on demographics, diagnoses, acute treatment, previous prophylactic treatments (including other CGRP antibodies), relevant comorbidities, mood disorders, and headache frequency. For the first three months of the treatment, we recorded data on headache frequency, acute treatment consumption, and adverse events. Institutional review board approval (Cantabria Ethics Committee, 28/2020, 11 December) and written informed consent were obtained from every patient.

### Statistical Analysis

Categorical variables are reported as percentages, whereas continuous variables are displayed as means ± SDs, together with medians and ranges unless stated differently in the text. The assumption of the normality of the quantitative variables was checked using the Shapiro–Wilk test. The Mann–Whitney U test was performed to assess the statistical differences between sub-groups in terms of the continuous variables. To evaluate the changes in the variables over time, Wilcoxon’s signed rank test was employed. For comparisons of categorical variables, Fisher’s exact test was carried out.

To identify independent predictors of a ≥50% response rate, an exploratory forward stepwise multiple logistic regression model was applied. Variables significantly associated with the response in the univariate analysis and clinically relevant variables were included as covariates.

The *p*-values presented are for two-tailed testing, and we considered *p* < 0.05 to prove significance. Estimated odds ratios (ORs) were reported together with their 95% confidence intervals (95% CIs). No methods for the imputation of missing values were used. All of the analyses were performed using SPSS version 28.0 software (SPSS Inc., Chicago, IL, USA).

## 3. Results

### 3.1. Sample Size, Demographics, and Baseline Characteristics

A total of 89 patients (all Caucasians) were prescribed fremanezumab during the year 2023 at our clinic, with all of them receiving a 225 mg dose monthly according to the clinician´s criteria. All of them consented to participation in this study. The mean age of the total cohort was 45.8 ± 12.5 (range: 18–81) years, and 77 (86.5%) of them were women.

**Figure 1:**
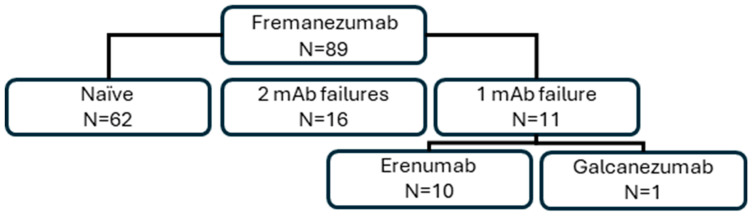
Flowchart of the study design. Of the 89 patients who started fremanezumab, 62 were naïve, 11 patients had received at least one monoclonal antibody previously, and 16 patients had received two different monoclonal antibodies.

Of the 89 participants, 23 (25.8%) met the HF criteria and 66 (74.2%) the CM criteria. Eleven (12.4%) reported aura. The average number of headache days for the three months before the start of the fremanezumab treatment was 21.1 ± 7.6 (range: 8–30 days). A total of 37 (41.6%) of the patients had experienced more than 28 headache days per month in the previous quarter. The patients who were treated with fremanezumab had remained in an HF/CM situation for an average of 8.1 ± 6.5 years (median: 6 years; range: 1–32 years). Medication overuse criteria were met by 53 (59.6%) patients. Four (4.5%) patients took opioids as an acute treatment; the rest either received NSAIDs and/or triptans. A total of 27 patients had previous experience with other CGRP antibodies; while 16 (18.0%) had used both erenumab and galcanezumab, 10 further patients had been treated only with erenumab, and 1 more patient had only received galcanezumab. Patients on erenumab or galcanezumab were switched to fremanezumab due to a lack of response after six months; no patient switched to fremanezumab due to experiencing adverse events due to taking erenumab or galcanezumab (Figure 1).

The most common comorbidities were anxiety–depression (42 patients; 47.2%) and fibromyalgia (22 patients; 24.7%) (Table 1).

### 3.2. Efficacy and Tolerability

The average number of headache days in the first three months of the fremanezumab treatment was 12.4 ± 11.2 (−8.7 ± 7.1 days compared to the quarter previous to fremanezumab use). This decrease was significant (*p* < 0.001). With regard to the reduction in headache days, 55 (61.8%) were ≥50% responders, and 24 (27.0%) were ≥75% responders, with just 2 being 100% responders. The rate of ≥50% and ≥75% responses in the patients naïve to CGRP antibodies was 75.8% (47/62) and 33.9% (21/62), respectively.

A total of 26 (29.2%) patients met the overuse criteria during the first quarter after the fremanezumab treatment. A total of 18 patients (20.2%) suffered from (mild) adverse events, and these are detailed in Table 1. There were no serious infusion reactions; only three patients had a minor skin rash at the injection site. No serious adverse events were recorded.

### 3.3. Factors Predicting Response

#### 3.3.1. The Univariate Analysis

We analyzed the influence of several clinical variables on the response to fremanezumab (Table 2). Age (≤40 years: 66.7%; >40 years: 60%; *p* = 0.630), the presence of aura (*p* = 0.105), and sex did not significantly modify the response to fremanezumab. Women had a numerically higher ≥50% response as compared to that in males, but these differences were not significant (63.6% versus 50.0%; *p* = 0.524).

**Figure 2:**
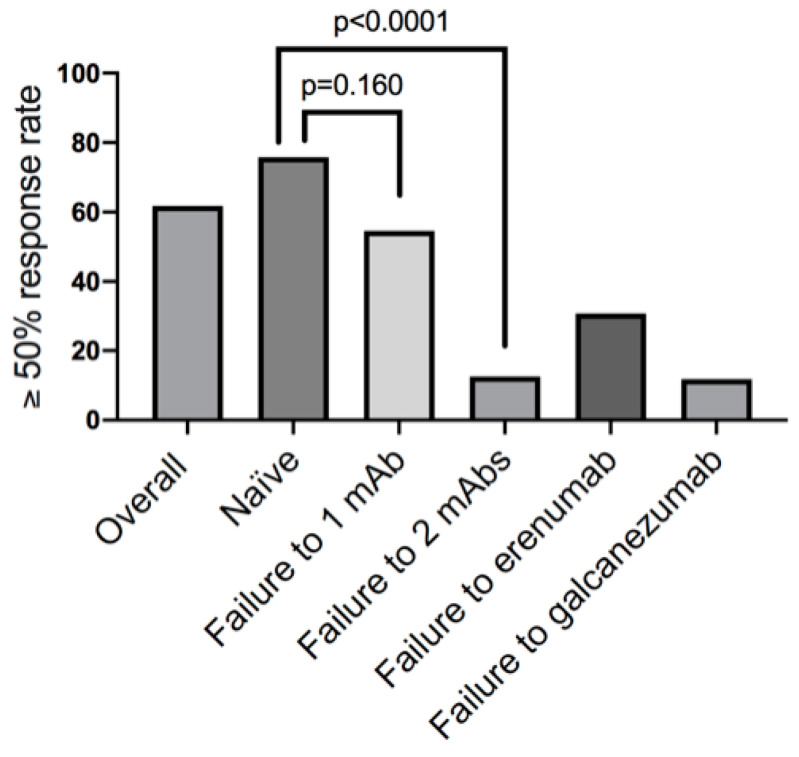
Overall ≥50% response rates of patients treated with fremanezumab in this series, as well as in naïve patients and classified by previous failure to respond to anti-CGRP monoclonal antibodies.

A total of 16 patients (18.0%) had been treated with both erenumab and galcanezumab without a response. The rate of a ≥50% response in these patients was 12.5% (2 out of 16). When compared to the response rate in naïve patients, these differences were significant (75.8%; 47 out of 62; *p* < 0.0001). Of our patients treated with fremanezumab, 11 (12.4%) had previously been treated with just one CGRP antibody (10 with erenumab and 1 with galcanezumab). In the erenumab group, six (60.0%) patients responded to fremanezumab, while the patient who previously failed to respond to galcanezumab did not respond to fremanezumab either. Therefore, in total, 17 and 26 patients had been treated at least with galcanezumab or erenumab, with their ≥50% response rates being 11.8% and 30.8%, respectively (Figure 2).

The HF/CM disease duration strongly influenced the response to fremanezumab. Patients with a ≥50% response to fremanezumab had remained in an HF/CM situation for an average of 5.8 ± 4.8 years (median: 5 years; range: 1–25 years) versus the average of 11.9 ± 7.1 years (median: 10 years; range: 2–32 years) in those with no response to fremanezumab (*p* < 0.0001). When classified between patients who had suffered from migraines for more than 6 years (the median value in our group) and those who had not, this latter group had a higher ≥50% response rate (82.0%) than the rate in the longer-duration group (35.9%; *p* < 0.0001).

Headache frequency had an effect on the response. First, the response to fremanezumab in the HF patients (22 out of 24; 91.7%) was better that in the CM patients (33 out of 65; 50.8%) (*p* < 0.001). Second, the patients without daily or almost daily headaches (≥28 days per month) had a higher ≥50% response to fremanezumab (48/58; 82.8%) than the response in those with almost daily headaches (7/31; 22.6%, *p* < 0.0001). Also, analgesic overuse was associated with a significantly lower response rate to fremanezumab (23 out of 53; 43.4%) versus that in non-abusers (32 out of 36; 88.9%; *p* < 0.0001).

Regarding the main comorbidities, the absence of anxiety–depression was associated with a higher ≥50% response rate (80.9%) compared to its presence (40.5%; *p* < 0.001). Also, patients without fibromyalgia had a higher ≥50% response rate (73.1%) than that in those who suffered from this condition (27.3%; *p* < 0.001).

#### 3.3.2. The Multivariate Analysis

**Figure 3:**
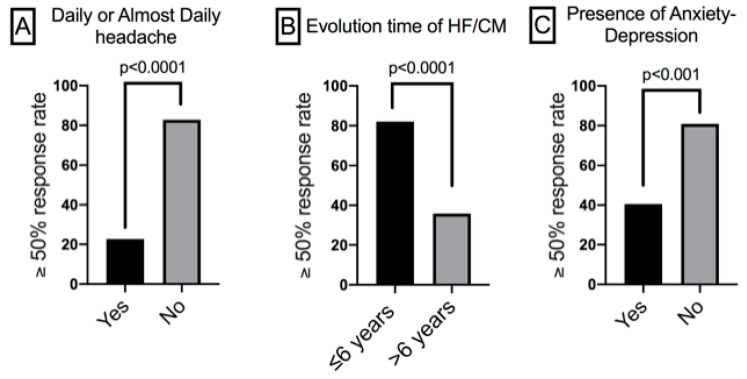
A univariate comparison of the ≥50% response rates of patients treated with fremanezumab classified by the parameters which remained significant after the forward stepwise multivariate logistic regression analysis. HF/CM: high-frequency episodic migraine/chronic migraine.

The results of the multivariate logistic analysis revealed that almost daily headaches (*p* < 0.001), a duration of HF/CM exceeding six years (*p* = 0.015), and anxiety–depression (*p* = 0.017) (Figure 3) were the remaining statistically significant variables. (Table 3).

The patients who responded were significantly younger than those who did not respond (43.3 ± 11.5 years vs. 49.8 ± 13.1 years; 0.018), but the patients who responded to fremanezumab had a significantly shorter CM/HF duration (5.8 ± 4.8 vs. 11.9 ± 7 years; *p* < 0.0001), and the age differences in the response disappeared in the multivariate analysis.

## 4. Discussion

These real-life results uphold the efficacy and security of fremanezumab, the third CGRP antibody available in our region, in refractory HF/CM patients even during the first quarter of treatment. In fact, the number of headache days was reduced by more than 50% in six out of ten patients, and there was a global reduction of 40% in the average number of headache days per month, and the rate of analgesic overuse fell from 58.6% to 26.8%. There were no serious adverse events, and its tolerability was excellent, with some constipation in around one out of eight fremanezumab-treated patients as the only complaint worth mentioning.

Our global results are in line with those coming from controlled clinical trials and real-world studies using fremanezumab [1,2,3,4,5,6,7,8,9,10,11,12] and other CGRP antibodies [15,16,20,21]. One intriguing aspect of all of these studies is that around 40% of patients with an HF/CM phenotype do not respond to CGRP antibodies. This lack of response has been ascribed potential explanations, such as the mistreatment of migraine mimics or the predominant role of other pain-producing neuropeptides in certain patients, but there is a need to try to identify the clinical profiles of migraine patients with a response and those without a response to CGRP antibodies. In general, previous real-world studies using CGRP antibodies have found a better response in patients with a purer migraine phenotype, those with associated allodynia, and those older than 40 years (15–20), while several laboratory works have reported a relationship (globally significant but not uniform for individual subjects) between elevated CGRP levels in the blood or saliva and the response to CGRP antibodies [22,23]. Our real-life data add further useful information for clinical practice in this regard. First, they confirm that a relevant proportion of patients (half in our experience) respond to fremanezumab after they have failed for at least sixth months to respond to erenumab, a CGRP antibody with a different target [11,24,25,26,27]. By contrast, the rate of the response to fremanezumab in patients who had failed to respond to both erenumab and galcanezumab was poor (12.5%) and significantly lower than that observed in the naïve HF/CM patients, who showed better response rates [28]. Interestingly, the response to fremanezumab in patients with previous failure to respond to erenumab was much better than that in those with a lack of response to galcanezumab. In other words, it seems sensible to try a switch from one CGRP antibody to another drug with a different mode of action [29], but trying a third CGRP antibody in patients with no consecutive response to both a receptor-targeted and a ligand-targeted CGRP antibody hardly seems justifiable on the basis of a cost-effectiveness analysis.

The univariate analysis showed that sex did not significantly influence the response to fremanezumab, which was in line with the results of previous real-world studies. Some studies have suggested a better response to CGRP antibodies in older patients [30,31], while Barbanti et al. found a higher response in young subjects [32]. In our work, we did not find a significant relationship between age and the response to fremanezumab, possibly because of the low numbers of young and old patients included here and also because the potential positive effect of older age on the response could be counteracted by the longer evolution of HF/CM.

Migraine frequency clearly influenced the fremanezumab response rate. The strongest factor predicting the response to fremanezumab was the presence of almost daily headaches (≥28 days—four complete weeks—per month), and the response was also significantly better in the HF patients versus that in the CM patients. Our results are in line with the findings of previous real-life studies using fremanezumab that analyzed this point [8,29,30,31]. Two potential explanations could account for these results. First, these patients do meet the CM criteria, as they have a history of episodic migraines and experience 15 or more days of migraine-like episodes per month, but in their current headaches, there could be a predominant tension type, a psychogenic element, or others, such as a cervicogenic component. Second, it is theoretically possible that these patients, with greater medication overuse and highly sensitized trigeminovascular systems and central pain pathways, are refractory or need more time (or higher dosages) to obtain a response.

The second strongest predictor of response was the HF/CM duration. Patients, mainly naïve, who had suffered from HF/CM for ≤6 years had a significantly higher probability of a response when compared to that in patients whose HF/CM had lasted longer than 6 years. Such data corroborate previous findings [32] and call for reconsideration of the reimbursement criteria for CGRP antagonists as an earlier preventive treatment to improve the likelihood of a response [33,34,35].

Finally, the response was also negatively influenced by the two main comorbidities suffered by our patients, anxiety–depression and/or fibromyalgia, though only the presence of anxiety–depression was actually associated with a poorer response after the multivariate analysis. Again, our results concur with those found in previous work [36], though the efficacy of fremanezumab in patients with anxiety–depression has been demonstrated in controlled [37] and real-life studies [38].

This real-world, prospective study avoids the constraints of randomized clinical trials and incorporates different outcomes and analyses but has several limitations, such as its rather small sample size, partly because of its prospective characteristics (in comparison with randomized controlled trials) and its single-center design; there is also no differentiation of different ethnicities and races and a short observation period. In addition, the clinical predictors analyzed here do not preclude the existence of others.

## 5. Conclusions

These real-world results confirm the efficacy of fremanezumab. Factors such as almost daily headaches and a headache history duration extending beyond six years were linked to the absence of a response. Around half of the patients who failed to respond to erenumab improved on fremanezumab, making a switch to a treatment with a new mode of action a reasonable move; however, our data suggest that routinely trying a third CGRP antibody in patients who have not responded to either a receptor-targeted or a ligand-targeted CGRP antibody may be challenging to justify.

## Figures and Tables

**Table 1:** Summary of demographic and clinical characteristics and response and tolerability data of the study group.

Baseline Characteristics	
Age (years): mean ± SD	45.8 ± 12.5
Sex (females): count (%)	77 (86.5)
Evolution time (years): mean ± SD	8.1 ± 6.5
Presence of aura (yes): count (%)	11 (12.4)
Basal monthly headache days: mean ± SD	21.1 ± 7.6
**Acute treatment**	
Medication overuse (yes): count (%)	53 (59.6)
Opioids (yes): count (%)	4 (4.5)
**Previous failure of a CGRP monoclonal antibody**	
Previous Failure Only of Erenumab (yes): count (%)	10 (11.2)
Previous Failure Only of Galcanezumab (yes): count (%)	1 (1.1)
Previous Failure of Erenumab and Galcanezumab (yes): count (%)	16 (18.0)
**Migraine classification**	
High-Frequency Migraine (yes): count (%)	23 (25.8)
>28 Basal Monthly Headache Days (yes): count (%)	37 (41.6)
**Comorbidities**	
Anxiety–depressive syndrome (yes): count (%)	42 (47.2)
Fibromyalgia (yes): count (%)	22 (24.7)
**Response variables after 3 months of treatment with fremanezumab**	
Reduction in Monthly Headache Days: mean ± SD	8.7 ± 7.1
≥50% Response Rate (yes): count (%)	55 (61.8)
≥75% Response Rate (yes): count (%)	24 (27.0)
Medication Overuse (yes): count (%)	26 (29.2)
**Adverse events after 3 months of treatment with fremanezumab**	
At least one (yes): count (%)	18 (20.2)
Constipation (yes): count (%)	11 (12.4)
Local reaction (yes): count (%)	3 (3.4)
Transient dizziness (yes): count (%)	2 (2.0)
Flu-like symptoms (yes): count (%)	2 (2.0)

**Table 2:** Univariate comparisons of ≥50% response rates among patients when classified by different clinical criteria.

Classification Criterion	Yes (% of ≥50% Response Rate)	No (% of ≥50% Response Rate)	*p*-Value
**Age ≤ 40 Years**	66.7	60.0	0.630
**Female**	63.6	50.0	0.524
**Presence of Aura**	81.8	59.0	0.105
**High Frequency**	91.7	50.8	<0.001
**Anxiety–Depression**	40.5	80.9	<0.001
**Fibromyalgia**	27.3	73.1	<0.001
**Basal Medication Overuse**	43.4	88.9	<0.0001
**>28 Basal Monthly Headache Days**	22.6	82.8	<0.0001
**HF/CM Length of ≤6 Years**	82.0	35.9	<0.0001

**Table 3:** Forward stepwise multivariate logistic regression analysis of independent predictors of ≥50% response rates. Variables shown are only those which were significantly associated. β: coefficient; SE: standard error; CI: confidence interval.

	β ± SE	Wald	*p*-Value	Odds Ratio	95% CI
**Anxiety–Depression (Yes vs. No)**	−1.495 ± 0.626	5.713	0.017	0.224	0.066–0.764
**>28 Basal Monthly Headache Days (Yes vs. No)**	−2.570 ± 0.629	16.675	<0.001	0.077	0.022–0.263
**Disease Duration (>6 Years vs. ≤6 Years)**	−1.485 ± 0.610	5.937	0.015	0.226	0.069–0.748

## Data Availability

The datasets used and/or analyzed during the current study are only available from the corresponding author, V.G.-Q. (vicente.gonzalez@scsalud.es), upon reasonable request, as they contain information that could compromise the privacy of the research participants.

## Abbreviations

The following abbreviations are used in this manuscript:

HF	High-frequency episodic migraine
CM	Chronic migraine
CGRP	Calcitonin-gene-related peptide
